# Assessment of the Clinical Trials Safety Profile of PD-1/PD-L1 Inhibitors Among Patients With Cancer: An Updated Systematic Review and Meta-Analysis

**DOI:** 10.3389/fonc.2021.662392

**Published:** 2021-05-24

**Authors:** Yuan Tian, Alan Huang, Yue Yang, Qi Dang, Qing Wen, Linlin Wang, Yuping Sun

**Affiliations:** ^1^ Special Needs Department of Proton Therapy Center, Shandong Cancer Hospital and Institute, Shandong First Medical University and Shandong Academy of Medical Sciences, Jinan, China; ^2^ Department of Oncology, Jinan Central Hospital, The First Hospital Affiliated with Shandong First Medical University, Jinan, China; ^3^ Human Resources Department, Jinan Central Hospital Affiliated to Shandong University, Jinan, China; ^4^ Jinan Clinical Research Center of Shandong First Medical University, Jinan, China; ^5^ Department of Radiation Oncology, Shandong Cancer Hospital and Institute, Shandong First Medical University and Shandong Academy of Medical Sciences, Jinan, China

**Keywords:** PD-1/PD-L1 inhibitors, cancer, meta-analysis, safety assessment, clinical trial

## Abstract

**Background:**

Understanding the safety and adverse event profiles of PD-1/PD-L1 inhibitors is important in guiding cancer immunotherapy. Consequently, we designed this meta-analysis to evaluate the safety of PD-1/PD-L1 inhibitors in clinical trials involving cancer patients.

**Methods:**

Four safety indicators comprising treatment-related adverse events, death, discontinuation of therapy and grades 3–5 adverse events were evaluated using the random effect model. The quality of enrolled trials was assessed using the Newcastle Ottawa Scale (NOS).

**Results:**

Forty-four clinical trials were included in the final meta-analysis. Compared with chemotherapy, the risk of death due to the use of PD-1/PD-L1 inhibitors was much lower than that experienced in the control group (OR = 0.65, 95%CI: [0.47, 0.91], I^2^ = 0%, Z = 2.52 (P = 0.01)). Similar observations were apparent regarding the other three indicators of safety and also when the use of PD-1/PD-L1 inhibitors alone is compared with the combined use of PD-1/PD-L1 and CTLA-4. When used together with chemotherapy, PD-1/PD-L1 inhibitors increased the incidence of the adverse events as compared to the use of chemotherapy alone. Increased risks for adverse events were also noticed with the use of PD-1/PD-L1 inhibitors over the use of a placebo.

**Conclusion:**

The use of PD-1/PD-L1 inhibitors alone is associated with a better safety profile compared to either the use of chemotherapy or the use of PD-1/PD-L1 inhibitors with other anticancer regimens.

## Introduction

Cancer immunotherapies, including immune checkpoint inhibitors (ICIs) and adoptive cell therapy, have revolutionized the treatment landscape and improved the survival prognosis for most cancer patients (1). Among these, PD-1/PD-L1 inhibitors are the most common type of immunosuppressants used in the treatment of solid tumors (1–4). PD-1/PD-L1 inhibitors can block the interaction between tumor cells and T cells, restore the immune recognition function of T cells, and then eliminate tumor cells (1–4). The unique anti-tumor mechanism of PD-1/PD-L1 inhibitors means that the toxicities caused by these agents are also different from other traditional anti-tumor drugs (1).

Although PD-1/PD-L1 inhibitors have shown remarkable clinical benefits in the treatment of tumors, the spectrum of immune-related adverse events (irAEs) that affect body organs are a major concern with the use of these agents (5, 6). Serious adverse events are a frequent limitation in the use of PD-1/PD-L1 inhibitors among cancer patients (5–9). It, therefore, behooves clinicians to conduct adequate and elaborate systematic assessment of potential recipients of these therapies, to ensure that the benefits outweigh the potential risks in the use of PD-1/PD-L1 inhibitors. In view of the limitations of previous meta-analyses regarding the safety and toxicity of PD-1/PD-L1 inhibitors (10–12), and the availability of recent information from results of clinical trials, we designed this study to reassess the safety of PD-1/PD-L1 inhibitors in cancer chemotherapy.

## Method

This study was performed according to the Preferred Reporting Items for Systematic Reviews and Meta-analyses (PRISMA) (13).

### Selection Criteria for Clinical Trials

All randomized, open-label, controlled clinical trials with efficacy and safety data of PD-1/PD-L1 inhibitors were explored. Although Phase III clinical trials were given priority, the phase of clinical trials was not the primary inclusion criterion. Malignancies were limited to solid tumors and, as such, hematological tumors were excluded from the study. The four safety indicators evaluated in the meta-analysis were: a) treatment-related death, b) treatment-related adverse events leading to discontinuation of therapy, c) treatment-related grades 3–5 adverse events and d) any treatment-related adverse events.

### Search Strategy

We followed the guidelines of the participants, interventions, comparisons, outcomes (PICOS) as recommended by the Cochrane Collaboration (13). A PubMed search was conducted using the search terms: “neoplasm”, “cancer”, “precancer”, “pre-cancer”, “malignant”, “premalignant”, “tumor”, “PD-1”, “PD-L1”, “nivolumab”, “Opdivo”, “pembrolizumab”, “Keytruda”, “Imfinzi”, “MK-3475”, “atezolizumab”, “Tecentriq”, “MPDL3280A”, “avelumab”, “Bavencio”, “durvalumab”, “camrelizumab”, and “BMS-963558”.

Articles were only included if they were published in English between 09 July 2013 and 19 Sep 2020. Three researchers were designated to independently scrutinize all the data and where there was duplication of clinical trials in selected articles, only one was used for the final analysis.

### Assessment of Study Quality and Publication Bias

The Cochrane Collaboration tool was used to assess risk of bias in randomized trials (14), while the Funnel plot and Egger’s test were applied to evaluate publication bias (15). Three researchers independently checked the quality of all the included clinical trials. The quality assessment comprised evaluating: a) Selection bias (random sequence generation and allocation concealment), b) Performance bias (blinding of participants and personnel), c) Detection bias (blinding of outcome assessment), d) Attrition bias (incomplete outcome data) and e) Reporting bias (selective outcome reporting).

### Outcome and Exposure of Interest

Our primary assessment indicators were the incidence of PD-1/PD-L1 inhibitors-induced “treatment-related death” and “treatment-related adverse events leading to discontinuation”. “Treatment-related grades 3-5 adverse events” and “any treatment-related adverse events” were also recorded. The basic characteristics and information on all the enrolled clinical trials were collected, including the first author’s name, year of publication, trial number, trial title, trial phase, the specific name of the anti-PD-1/PD-L1 agent, treatment regimens, whether treatment was first-line or not, tumor types and the number of participants, treatment-related death and treatment-related discontinuation.

### Assessment of Heterogeneity and Statistical Analysis

Heterogeneity of all the eligible trials was evaluated using Cochrane’s Q statistic and the I^2^ statistic as reported by Higgins and colleagues (13, 16). Publication bias was checked using the Harbord test (16). Using the I^2^ value, heterogeneity was regarded as low (<25%), moderate (25–50%) or high (>50%). Odds ratio (OR) and the corresponding 95% confidence interval (CI) were calculated using the random effect (RE) (17). Data analysis was conducted using Review Manager 5.3 and all statistical tests were two-sided with a value of *P <*0.05 considered statistically significant. Subgroup analysis was performed according to the tumor type, treatment regimen and PD-1/PD-L1 inhibitor used.

## Results

### Literature Search Results

**Figure 1:**
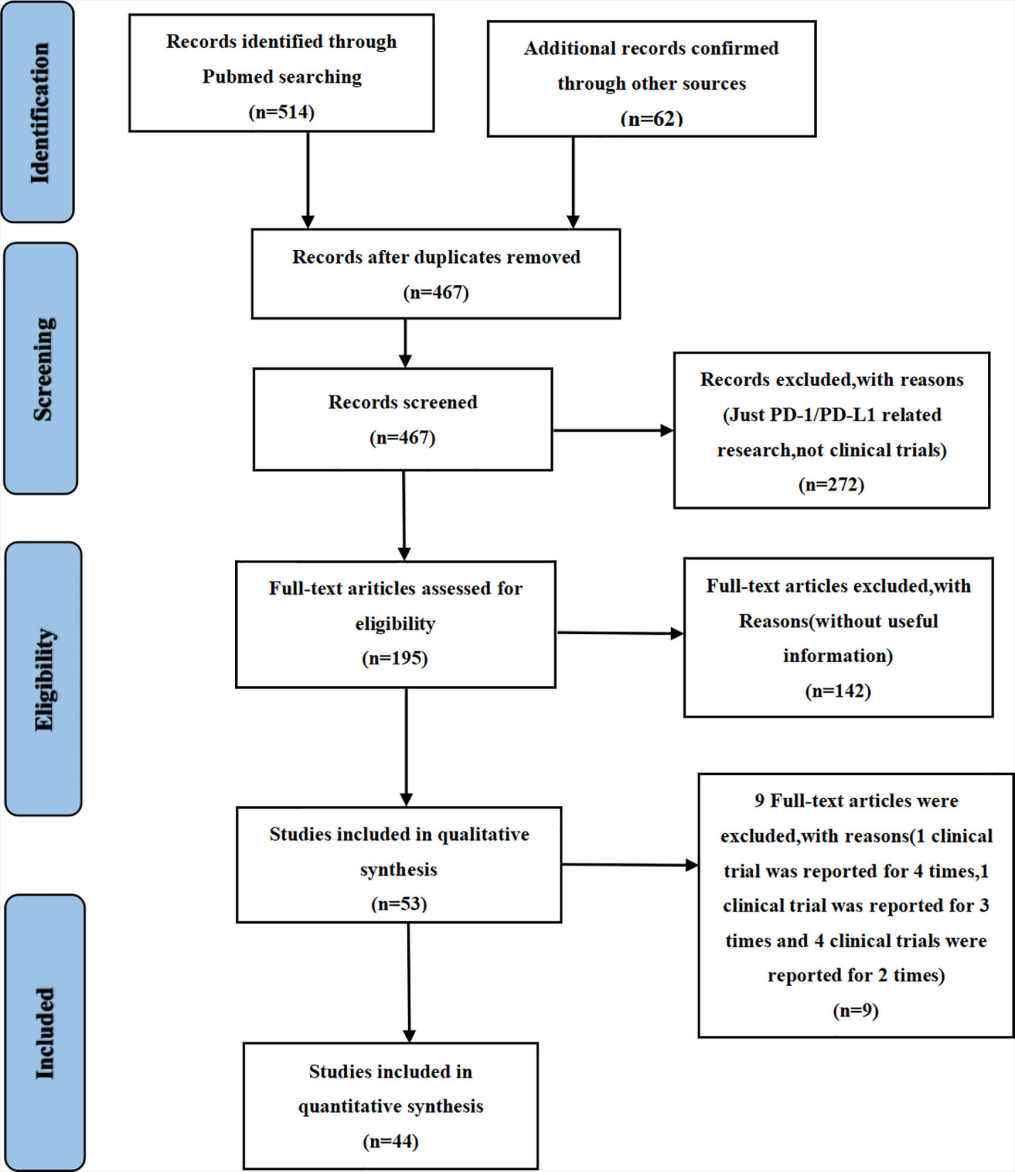
The flow diagram of enrolled clinical trials.

**Figure 2:**
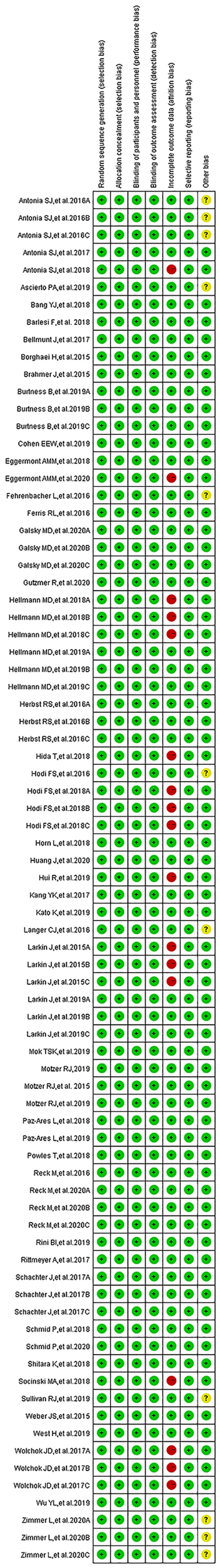
Risk of bias summary.

We found 514 clinical trials investigating PD-1/PD-L1 inhibitors after conducting an initial search through the PubMed website. Fifty-three articles were deemed to meet our preliminary selection criteria (18–70), of which 44 articles were selected for the final comprehensive analysis (18–21, 23–30, 32–42, 44–50, 52, 53, 56, 57, 61–70). The results of 6 clinical trials had been reported in multiple platforms: CheckMate 067 (n = 4) (57–60), PACIFIC (n = 3) (54–56), CheckMate 227 (n = 2) (21, 22), OAK (n = 2) (31, 32), KeyNote 054 (n = 2) (51, 52) and IMpower 150 (n = 2) (42, 43). When such duplications were noted, only one was selected for the meta-analysis. The PRISMA flow diagram of the screening process for the clinical trials is shown in **Figure 1** while the quality assessment of included studies is shown in **Figure 2**.

### Characteristics of Clinical Trials

The basic characteristics of the 53 eligible articles are summarized in **Table 1** (18–70). Most of the articles (45) were about phase III clinical trials (18–35, 38–48, 51–60, 63, 64, 66, 68–70), while five were phase II trials (37, 49, 50, 62, 65). The rest were a phase I trial (67), a phase I/II trial (61), and a phase II/III trial (36). As shown in **Table 1**, 28 clinical trials (reported in 33 articles) were associated with PD-1 inhibitors (18, 20–26, 28, 33–36, 38–41, 46, 49–53, 57–63, 65, 66, 70), while the other 16 clinical trials (reported in 20 articles) were associated with PD-L1 inhibitors (19, 27, 29–32, 37, 42–45, 47, 48, 54–56, 64, 67–69). Nivolumab (14 clinical trials) (20–22, 24, 35, 38–40, 50, 53, 57–62, 66, 70), Pembrolizumab (13 clinical trials) (23, 25, 26, 28, 33, 34, 36, 41, 46, 49, 51, 52, 63, 65), and atezolizumab (11 clinical trials) (19, 27, 31, 32, 37, 42–44, 47, 48, 64, 67, 69), were the most reported PD-1/PD-L1 inhibitors. Fewer studies involved Camrelizumab (18), Durvalumab (45, 54–56), and Avelumab (29, 30, 68).

**Table 1:** Baseline characteristics of included articles.

No.	Reference	NCT number	Phase	Drug name	Treatment regimen	First-line treatment	Tumor type	Involving patients	Treatment related deaths	Treatment related discontinuation	Treatment related grades 3–5 adverse events	Treatment related any adverse events
PD-1/PD-L1 *VS.* Chemotherapy												
11	Huang et al. (18)	NCT03099382 (ESCORT)	III	Camrelizumab (PD-1)	Camrelizumab *VS.* (Docetaxel, irinotecan)	NO	OSCC	448	10	28	131	413
22	Galsky et al. (19)	NCT02807636 (IMvigor130)	III	Atezolizumab (PD-L1)	Atezolizumab *VS.* GC	YES	UC	744	7	N/A	376	584
33	Kato et al. (20)	NCT02569242 (ATTRACTION-3)	III	Nivolumab (PD-1)	Nivolumab *VS.* (Paclitaxel or Docetaxel)	NO	OSCC	417	5	37	171	335
44	Hellmann et al. (21)	NCT02477826 (CheckMate227)	III	Nivolumab (PD-1)	Nivolumab *VS.* Platinum doublet Chemotherapy	YES	NSCLC	961	8	100	281	723
	Hellmann et al. (22)									96	280	711
55	Mok et al. (23)	NCT02220894 (KEYNOTE-042)	III	Pembrolizumab (PD-1)	Pembrolizumab *VS.* PC or CP	YES	NSCLC	1,251	27	115	365	952
66	Wu et al. (24)	NCT02613507 (CheckMate078)	III	Nivolumab (PD-1)	Nivolumab *VS.* Docetaxel	NO	NSCLC	493	7	27	109	346
77	Cohen et al. (25)	NCT02252042 (KEYNOTE-040)	III	Pembrolizumab (PD-1)	Pembrolizumab *VS.* (Methotrexate, Docetaxel or Cetuximab)	NO	HNSCC	480	6	27	118	351
821	Burtness et al. (26)	NCT02358031 (KEYNOTE-048)	III	Pembrolizumab (PD-1)	Pembrolizumab *VS.* Cetuximab + Chemotherapy	YES	HNSCC	587	11	N/A	250	453
98	Powles et al. (27)	NCT02302807 (IMvigor211)	III	Atezolizumab (PD-L1)	Atezolizumab *VS.* Chemotherapy	NO	UC	902	13	79	293	714
109	Shitara et al. (28)	NCT02370498 (KEYNOTE-061)	III	Pembrolizumab (PD-1)	Pembrolizumab *VS.* Paclitaxel	NO	GC/GEJC	570	4	24	138	387
1110	Barlesi et al. (29)	NCT02395172 (JAVELIN Lung 200)	III	Avelumab (PD-L1)	Avelumab *VS.* Docetaxel	NO	NSCLC	758	18	79	219	564
1211	Bang et al. (30)	NCT02625623 (JAVELINGastric300)	III	Avelumab (PD-L1)	Avelumab *VS.* Paclitaxel or Irinotecan	NO	GC/GEJC	361	1	16	73	221
1312	Hida et al. (31)	NCT02008227 (OAK)	III	Atezolizumab (PD-L1)	Atezolizumab *VS.* Docetaxel	NO	NSCLC	101	0	13	54	93
	Rittmeyer et al. (32)							1,187	1	N/A	337	886
1413	Bellmunt et al. (33)	NCT02256436 (KEYNOTE-045)	III	Pembrolizumab (PD-1)	Pembrolizumab *VS.* (Paclitaxel, Docetaxel, or Vinflunine)	NO	UC	521	8	43	166	392
1514	Reck et al. (34)	NCT02142738 (KEYNOTE-024)	III	Pembrolizumab (PD-1)	Pembrolizumab *VS.* Platinum-based Chemotherapy	YES	NSCLC	304	4	27	121	248
1615	Ferris et al. (35)	NCT02105636 (CheckMate141)	III	Nivolumab (PD-1)	Nivolumab *VS.* (Methotrexate, Docetaxel, or Cetuximab)	NO	HNSCC	347	3	N/A	73	225
1716	Herbst et al. (36)	NCT01905657 (KEYNOTE-010)	II/III	Pembrolizumab (PD-1)	Pembrolizumab 2 mg/kg *VS.* Docetaxel	NO	NSCLC	648	8	46	152	466
	Herbst et al. (36)				Pembrolizumab 10 mg/kg *VS.* Docetaxel	NO	NSCLC	652	8	48	164	477
1817	Fehrenbacher et al. (37)	NCT01903993 (POPLAR)	II	Atezolizumab (PD-L1)	Atezolizumab *VS.* Docetaxel	NO	NSCLC	277	4	26	72	214
1918	Borghaei et al. (38)	NCT01673867 (CheckMate057)	III	Nivolumab (PD-1)	Nivolumab *VS.* Docetaxel	NO	NSCLC	555	2	54	174	435
2019	Brahmer et al. (39)	NCT01642004 (CheckMate017)	III	Nivolumab (PD-1)	Nivolumab *VS.* Docetaxel	NO	NSCLC	260	3	17	83	187
2120	Weber et al. (40)	NCT01721746 (CheckMate037)	III	Nivolumab (PD-1)	Nivolumab *VS.* (Dacarbazine or Paclitaxel + Carboplatin)	NO	Melanoma	370	0	14	56	262
PD-1/PD-L1 + Chemotherapy *VS.* Chemotherapy												
11	Schmid et al. (41)	NCT03036488 (KEYNOTE-522)	III	Pembrolizumab (PD-1)	Pembrolizumab + PC *VS.* PC	YES	TNBC	1,170	4	230	881	1161
22	Galsky et al. (19)	NCT02807636 (IMvigor130)	III	Atezolizumab (PD-L1)	Atezolizumab + GC *VS.* GC	YES	UC	843	13	N/A	695	807
3	Reck et al. (42)	NCT02366143 (IMpower150)	III	Atezolizumab (PD-L1)	Atezolizumab + BCP *VS.* BCP	YES	NSCLC	787	N/A	N/A	382	737
	Socinski et al. (43)								20	N/A	427	747
43	West et al. (44)	NCT02367781 (IMpower130)	III	Atezolizumab (PD-L1)	Atezolizumab + nPC *VS.* nPC	YES	NSCLC	705	9	N/A	495	670
54	Paz-Ares et al. (45)	NCT03043872 (CASPIAN)	III	Durvalumab (PD-L1)	Durvalumab + EP *VS.* EP	YES	SCLC	531	7	28	259	477
66	Paz-Ares et al. (46)	NCT02775435 (KEYNOTE-407)	III	Pembrolizumab (PD-1)	Pembrolizumab + PC or nPC *VS.* PC or nPC	YES	NSCLC	558	16	N/A	N/A	N/A
77	Horn et al. (47)	NCT02763579 (IMpower133)	III	Atezolizumab (PD-L1)	Atezolizumab + EC *VS.* EC	YES	SCLC	394	6	N/A	228	369
88	Schmid et al. (48)	NCT02425891 (IMpassion130)	III	Atezolizumab (PD-L1)	Atezolizumab + nab-Paclitaxel *VS.* nab-Paclitaxel	YES	TNBC	890	4	N/A	315	846
99	Langer et al. (49)	NCT02039674 (KEYNOTE-021)	III	Pembrolizumab (PD-1)	Pembrolizumab + CP *VS.* CP	YES	NSCLC	121	3	14	39	111
PD-1/PD-L1 *VS.* Placebo												
1	Zimmer et al. (50)	NCT02523313 (IMMUNED)	III	Nivolumab (PD-1)	Nivolumab *VS.* Placebo	NO	Melanoma	107	0	8	18	75
2	Eggermont et al. (51)	NCT02362594 (KEYNOTE-054)	III	Pembrolizumab (PD-1)	Pembrolizumab *VS.* Placebo	NO	Melanoma	1,011	2	N/A	91	731
3	Eggermont et al. (52)								1	74	92	728
4	Kang et al. (53)	NCT02267343 (ATTRACTION-2)	III	Nivolumab (PD-1)	Nivolumab *VS.* Placebo	NO	GC/GEJC	491	7	13	41	184
5	Hui et al. (54)	NCT02125461 (PACIFIC)	III	Durvalumab (PD-L1)	Durvalumab *VS.* Placebo	NO	NSCLC	709	N/A	N/A	N/A	N/A
6	Antonia et al. (55)										N/A	N/A
7	Antonia et al. (56)										76	447
PD-1 *VS.* PD-1 + CTLA-4												
1	Zimmer et al. (50)	NCT02523313 (IMMUNED)	III	Nivolumab (PD-1)	Nivolumab *VS.* Nivolumab + Ipilimumab	NO	Melanoma	111	0	41	54	100
2	Larkin et al. (57)	NCT01844505 (CheckMate067)	III	Nivolumab (PD-1)	Nivolumab *VS.* Nivolumab + Ipilimumab	YES	Melanoma	626	3	170	259	571
3	Hodi et al. (58)								3	165	255	570
4	Wolchok et al. (59)								3	160	251	570
5	Larkin et al. (60)								1	138	223	556
6	Hellmann et al. (21)	NCT02477826 (CheckMate227)	III	Nivolumab (PD-1)	Nivolumab *VS.* Nivolumab + Ipilimumab	YES	NSCLC	967	10	152	265	698
7	Hellmann et al. (22)								9	145	254	684
8	Antonia et al. (61)	NCT01928394 (CheckMate032)	I/II	Nivolumab (PD-1)	Nivolumab 3 mg/kg *VS.* Nivolumab 1 mg/kg + Ipilimumab 3 mg/kg	NO	SCLC	159	2	13	33	102
9	Antonia et al. (61)				Nivolumab 3 mg/kg *VS.* Nivolumab 3 mg/kg + ipilimumab1 mg/kg			152	1	10	24	93
PD-1+CTLA-4 *VS.* CTLA-4												
1	Larkin et al. (57)	NCT01844505 (CheckMate067)	III	Nivolumab (PD-1)	Nivolumab + Ipilimumab *VS.* Ipilimumab	YES	Melanoma	624	3	177	272	568
2	Hodi et al. (58)								3	173	272	567
3	Wolchok et al. (59)								3	172	270	568
4	Larkin et al. (60)								1	160	257	567
5	Hodi et al. (62)	NCT01927419 (CheckMate069)	II	Nivolumab (PD-1)	Nivolumab + Ipilimumab *VS.* Ipilimumab	YES	Melanoma	140	3	32	61	129
PD-1 *VS.* CTLA-4												
1	Larkin et al. (57)	NCT01844505 (CheckMate067)	III	Nivolumab (PD-1)	Nivolumab + Placebo *VS.* Ipilimumab	YES	Melanoma	624	2	87	159	539
2	Hodi et al. (58)									86	157	538
3	Wolchok et al. (59)									86	153	538
4	Larkin et al. (60)									70	136	525
5	Schachter et al. (63)	NCT01866319 (KEYNOTE-006)	III	Pembrolizumab (PD-1)	Pembrolizumab every 2 weeks *VS.* Ipilimumab	NO	Melanoma	534	1	42	97	419
	Schachter et al. (63)				Pembrolizumab every 3 weeks *VS.* Ipilimumab			533	0	53	96	403
PD-1/PD-L1 *VS.* PD-1/PD-L1 + Chemotherapy												
1	Galsky et al. (19)	NCT02807636 (IMvigor130)	III	Atezolizumab (PD-L1)	Atezolizumab *VS.* Atezolizumab + GC	YES	UC	807	12	N/A	433	645
2	Burtness et al. (26)	NCT02358031 (KEYNOTE-048)	III	Pembrolizumab (PD-1)	Pembrolizumab *VS.* Pembrolizumab + Chemotherapy	YES	HNSCC	576	14	N/A	249	439
Others												
11	Reck et al. (42)	NCT02366143 (IMpower150)	III	Atezolizumab (PD-L1)	ACP *VS.* ABCP	YES	NSCLC	793	N/A	N/A	364	727
	Reck et al. (42)				Atezolizumab + PC *VS.* Bevacizumab + PC			794	N/A	N/A	344	740
23	Zimmer et al. (50)	NCT02523313 (IMMUNED)	II	Nivolumab (PD-1)	Nivolumab + Ipilimumab *VS.* Placebo	NO	Melanoma	106	0	35	42	81
3	Gutzmer et al. (64)	NCT02908672 (IMspire150)	III	Atezolizumab (PD-L1)	Atezolizumab + VC *VS.* VC	YES	Melanoma	511	N/A	73	390	507
4	Ascierto et al. (65)	NCT02130466 (KEYNOTE-022)	II	Pembrolizumab (PD-1)	Pembrolizumab + DT *VS.* DT	NO	Melanoma	120	1	39	51	113
55	Motzer et al. (66)	NCT02231749 (CheckMate214)	III	Nivolumab (PD-1)	Nivolumab + Ipilimumab *VS.* Sunitinib	YES	RCC	1,082	12	185	599	1,034
6	Burtness et al. (26)	NCT02358031 (KEYNOTE-048)	III	Pembrolizumab (PD-1)	Pembrolizumab + Chemotherapy *VS.* Cetuximab + Chemotherapy	YES	HNSCC	563	19	N/A	397	542
77	Sullivan et al. (67)	NCT01656642	I	Atezolizumab (PD-L1)	Atezolizumab + vemurafenib *VS.* Atezolizumab + VC	YES	Melanoma	56	N/A	13	41	56
88	Hellmann et al. (21)	NCT02477826 (CheckMate227)	III	Nivolumab (PD-1)	Nivolumab + Ipilimumab *VS.* Platinum doublet Chemotherapy	YES	NSCLC	1,146	14	156	394	909
99	Hellmann et al. (22)								13	151	386	893
1010	Motzer et al. (68)	NCT02684006 (JAVELIN Renal 101)	III	Avelumab (PD-L1)	Avelumab + Axitinib *VS.* Sunitinib	YES	RCC	873	4	92	489	837
1111	Rini et al. (69)	NCT02420821 (IMmotion151)	III	Atezolizumab (PD-L1)	Atezolizumab + Bevacizumab *VS.* Sunitinib	YES	RCC	897	6	61	422	840
1212	Schachter et al. (63)	NCT01866319 (KEYNOTE-006)	III	Pembrolizumab (PD-1)	Pembrolizumab every 2 weeks *VS.* Pembrolizumab every 3 weeks	NO	Melanoma	555	1	49	93	442
1313	Antonia et al. (61)	NCT01928394 (CheckMate032)	I/II	Nivolumab (PD-1)	Nivolumab 1 mg/kg + Ipilimumab 3 mg/kg *VS.* Nivolumab 3 mg/kg + ipilimumab 1 mg/kg	NO	SCLC	115	3	11	31	91
1414	Herbst et al. (36)	NCT01905657 (KEYNOTE-010)	II/III	Pembrolizumab (PD-1)	Pembrolizumab 2 mg/kg *VS.* Pembrolizumab 10 mg/kg	NO	NSCLC	682	6	32	98	441
1515	Motzer et al. (70)	NCT01668784 (CheckMate025)	III	Nivolumab (PD-1)	Nivolumab *VS.* Everolimus	NO	RCC	803	2	83	221	668

PD-1, Programmed Cell Death-1; PD-L1, Programmed Cell Death Ligand 1; CTLA-4, Cytotoxic T lymphocyte associate protein-4; OSCC, Oesophageal Squamous Cell Carcinoma; UC, Urothelial Cancer; NSCLC, Non-Small Cell Lung Cancer; HNSCC, Head and Neck

Squamous Cell Carcinoma; GC/GEJC, Gastric or Gastro-oesophageal Junction Cancer; TNBC, Triple-negative Breast Cancer; SCLC, Small Cell Lung Cancer; HCC, Hepatocellular Carcinoma; RCC, Renal Cell Carcinoma; PC, Paclitaxel + Carboplatin; GC, Gemcitabine + Carboplatin/Cisplatin; BCP, Bevacizumab + Carboplatin + Paclitaxel; EP, Etoposide + Platinum; EC, Etoposide + Carboplatin, CP, Carboplatin + Pemetrexed; nPC, nab-Paclitaxel + Carboplatin; ACP, Atezolizumab + Carboplatin + Paclitaxel; ABCP, Atezolizumab + Bevacizumab + Carboplatin + Paclitaxel; VC, Vemurafenib + Cobimetinib; DT, Dabrafenib + Trametinib.

There were nine different types of tumors in all the recruited clinical trials. Most of these were non-small cell lung cancer (NSCLC) (15) (21–24, 29, 31, 32, 34, 36–39, 42–44, 46, 49, 54–56), and melanoma (9) (40, 50–52, 57–60, 62–65, 67). The other tumors included renal cell carcinoma (RCC) (4) (66, 68–70), urothelial cancer (UC) (3) (19, 27, 33), head and neck squamous cell carcinoma (HNSCC) (3) (25, 26, 35), and gastric or esophageal junction cancer (GC/GEJC) (3) (28, 30, 53). PD-1/PD-L1 inhibitors were prescribed as the first-line treatment regimen in 20 clinical trials (19, 21–23, 26, 34, 41–49, 57–60, 62, 64, 66–69), while previous anti-cancer treatments were found in 24 clinical trials (18, 20, 24, 25, 27–33, 35–40, 50–56, 61, 63, 65, 70).

The clinical trials were further stratified into seven groups according to the treatment regimen as shown in **Table 1**. The classes are Group A (PD-1/PD-L1 *vs* Chemotherapy) (18–40), Group B (PD-1/PD-L1 + Chemotherapy vs Chemotherapy) (19, 41–49), Group C (PD-1/PD-L1 *vs* Placebo) (50–56), Group D (PD-1 *vs* PD-1+CTLA-4) (21, 22, 50, 57–61), Group E (PD-1+CTLA-4 *vs* CTLA-4) (57–60, 62), Group F (PD-1 *vs* CTLA-4) (57–60, 63), and Group G (PD-1/PD-L1 *vs* PD-1/PD-L1 + Chemotherapy) (19, 26). The risks for the various types of adverse events within each group were then evaluated.

### Risk of Bias

The funnel plots assessing publication bias are as shown in the **Supplementary Figures S2–S6**. Other types of bias involving the 53 articles are summarized in **Figure 2**. Six clinical trials were associated with unclear risk of bias while high risk of bias (37, 50, 61, 62, 65, 67), mainly due to incomplete outcome data, hence attrition bias, was found with seven clinical trials (22, 31, 43, 51, 54, 55, 58–60).

### Incidence of Treatment-Related Death

**Figure 3:**
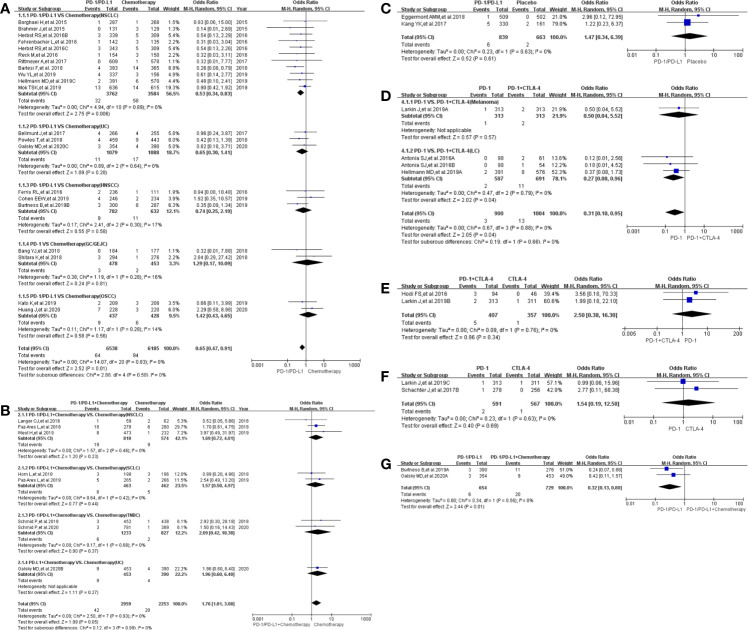
Forest plots of treatment-related adverse events leading to death. **(A)** The odds ratio of treatment-related adverse events leading to death calculated by the random effect (RE) model in Group A (PD-1/PD-L1 *vs* Chemotherapy). Subgroup analysis was performed based on tumor types. **(B)** The odds ratio of treatment-related adverse events leading to death calculated by the random effect (RE) model in Group B (PD-1/PD-L1 + Chemotherapy *vs* Chemotherapy). Subgroup analysis was performed based on tumor types. **(C)** The odds ratio of treatment-related adverse events leading to death calculated by the random effect (RE) model in Group C (PD-1/PD-L1 *vs* Placebo). **(D)** The odds ratio of treatment-related adverse events leading to death calculated by the random effect (RE) model in Group D (PD-1 *vs* PD-1 + CTLA-4). Subgroup analysis was performed based on tumor types. **(E)** The odds ratio of treatment-related adverse events leading to death calculated by the random effect (RE) model in Group E (PD-1 + CTLA-4 *vs* CTLA-4). **(F)** The odds ratio of treatment-related adverse events leading to death calculated by the random effect (RE) model in Group F (PD-1 *vs* CTLA-4). **(G)** The odds ratio of treatment-related adverse events leading to death calculated by the random effect (RE) model in Group G (PD-1/PD-L1 *vs* PD-1/PD-L1 + Chemotherapy).

Treatment-related death in studies comparing the use of PD-1/PD-L1 and chemotherapy (Group A) was reported in 21 clinical trials (18–40). Less deaths were reported in the PD-1/PD-L1 group as compared to the control chemotherapy group (OR = 0.65, 95%CI: [0.47, 0.91], I^2^ = 0%, Z = 2.52 (P = 0.01) (**Figure 3A**) (18–40). This observation was more evident with the NSCLC subgroup (OR = 0.53, 95% CI:[0.34, 0.83], I^2^ = 0%, Z = 2.75 (P = 0.006) (**Figure 3A**). In addition to a lack of heterogeneity between the groups (I^2^ = 0%) the funnel plots revealed that there was no obvious publication bias (**Supplementary Figure S3A**). Upon subgroup stratification this trend was more obvious with the PD-L1 related subgroup [OR = 0.39, 95% CI:(0.20, 0.74); **Supplementary Figures S1A** and **Figure S2A**]. Furthermore, we found that the risk of death in the PD-L1-related subgroup was lower than that in the PD-1-related subgroup [OR (0.39 *VS.* 0.78); P = 0.07, **Supplementary Figure S1A**]. Similar trends of treatment-related death were found in Group D (**Figure 3D**; **Supplementary Figure S3D**) and Group G (**Figure 3G**; **Supplementary Figure S3G**), when PD-1/PD-L1 inhibitors were compared with either PD-1 + CTLA-4 or PD-1/PD-L1 + Chemotherapy (19, 21, 22, 26, 50, 57–61).

When PD-1/PD-L1 inhibitors were prescribed in combination with chemotherapy, the risk of death was increased [OR = 1.76, 95%CI:(1.01, 3.08), I^2^ = 0%, Z = 1.99 (P = 0.05) (**Figure 3B**)] (19, 41, 44–49). Similar risk trends, although not statistically significant, were observed for the other Groups: Group C (**Figure 3C** **)**, Group E (**Figure 3E**) and Group F (**Figure 3F**) (50–60, 62, 63). The corresponding funnel plot analyses confirmed that there were no obvious publication bias (**Supplementary Figures S3B, C, E, F**).

### Incidence of Treatment-Related Adverse Events Leading to Discontinuation

**Figure 4:**
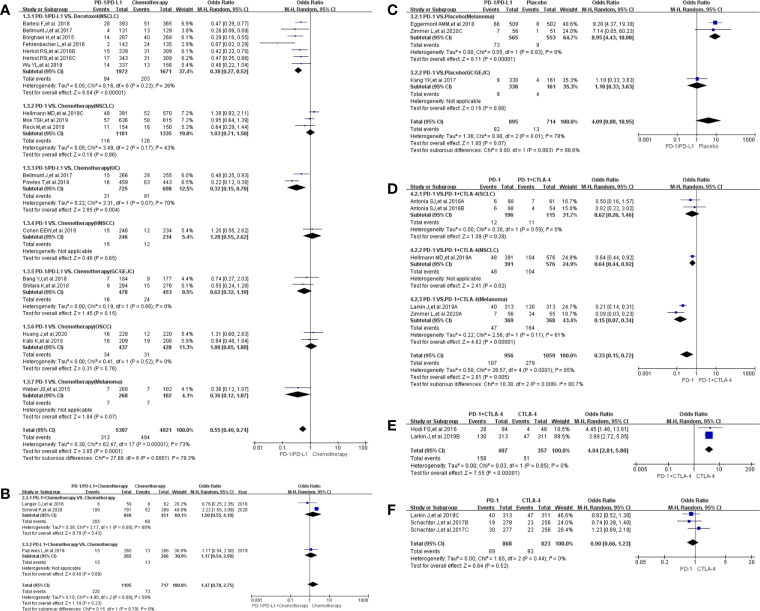
Forest plots of treatment-related adverse events leading to discontinuation of therapy. **(A)** The odds ratio of treatment-related adverse events leading to discontinuation of therapy calculated by the random effect (RE) model in Group A (PD-1/PD-L1 *vs* Chemotherapy): subgroup analysis was performed based on tumor types, PD-1/PD-L1 and treatment regimens. **(B)** The odds ratio of treatment-related adverse events leading to death calculated by the random effect (RE) model in Group B (PD-1/PD-L1 + Chemotherapy *vs* Chemotherapy). Subgroup analysis was performed based on PD-1/PD-L1. **(C)** The odds ratio of treatment-related adverse events leading to discontinuation of therapy calculated by the random effect (RE) model in Group C (PD-1/PD-L1 *vs* Placebo). Subgroup analysis was performed based on tumor types. **(D)** The odds ratio of treatment-related adverse events leading to discontinuation calculated by the random effect (RE) model in Group D (PD-1 *vs* PD-1 + CTLA-4). Subgroup analysis was performed based on tumor types. **(E)** The odds ratio of treatment-related adverse events leading to discontinuation of therapy calculated by the random effect (RE) model in Group E (PD-1 + CTLA-4 *vs* CTLA-4). **(F)** The odds ratio of treatment-related adverse events leading to discontinuation of therapy calculated by the random effect (RE) model in Group F (PD-1 *vs* CTLA-4).

The risk of treatment-related adverse events leading to discontinuation of therapy in the use of PD-1/PD-L1 was significantly lower than witnessed with the group that received chemotherapy [OR = 0.55, 95%CI:(0.40, 0.75), I^2^ = 77%, Z = 3.79 (P = 0.0001); **Figure 4A**] (18, 20, 22–25, 27–30, 33, 34, 36–40). Subgroup analysis showed that the risk of such adverse events was lower with the PD-L1-related subgroup as compared to the PD-1-related subgroup [OR (0.39 *vs.* 0.64); P = 0.15, **Supplementary Figure S1B**] (18, 20, 22–25, 27–30, 33, 34, 36–40). We also found high heterogeneity (I^2^ = 73%, **Figure 4A** and **Supplementary Figure S1B**) but no obvious publication bias (**Supplementary Figure S4A**). This trend is replicated when the use of PD-1 is compared with combined use of PD-1 plus CTLA-4, in Group D [OR = 0.33, 95%CI: (0.15, 0.72), I^2^ = 85%, Z = 2.81(P = 0.005); **Figure 4D**; **Supplementary Figure S4D**] (21, 50, 57, 61). However, a dissimilar trend was evident when PD-1 combined with CTLA-4 was compared with CTLA-4 alone, in Group E [OR = 4.04, 95%CI:(2.81, 5.80), I^2^ = 0%, Z = 7.55(P <0.00001); **Figure 4E**] (57, 62). Additional subgroup analyses did not yield statistically different results (**Figures 4B, C, F**) (41, 45, 49, 50, 52, 53, 57, 63).

### Incidence of Any Treatment-Related Adverse Events

**Figure 5:**
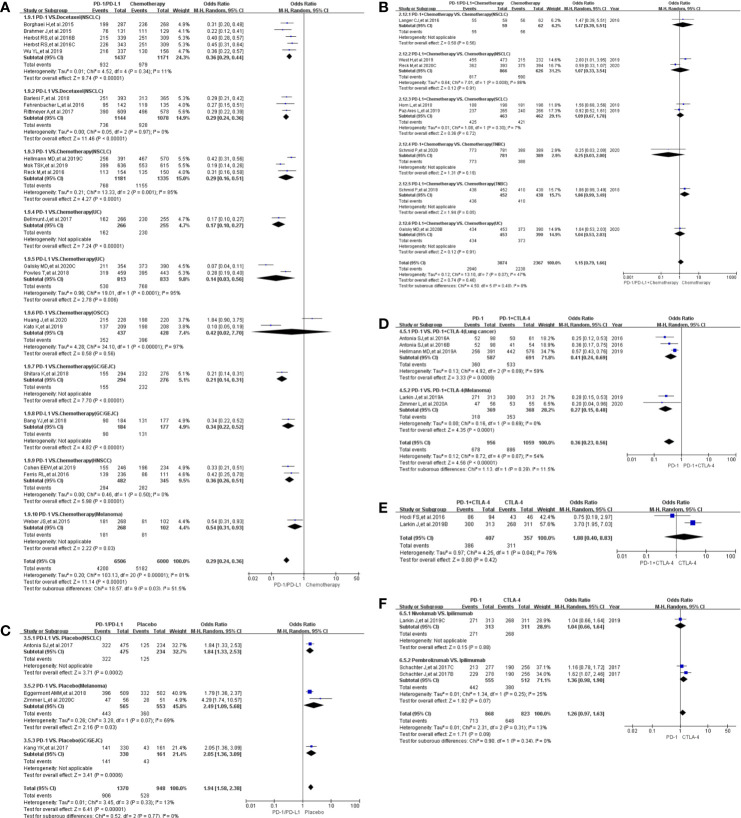
Forest plots of all-grade treatment-related adverse events. **(A)** The odds ratio of all-grade treatment-related adverse events calculated by the random effect (RE) model in Group A (PD-1/PD-L1 *vs* Chemotherapy). Subgroup analysis was performed based on tumor types, PD-1/PD-L1 and treatment regimens. **(B)** The odds ratio of all-grade treatment-related adverse events calculated by the random effect (RE) model in Group B (PD-1/PD-L1 + Chemotherapy *vs* Chemotherapy). Subgroup analysis was performed based on PD-1/PD-L1 and tumor types. **(C)** The odds ratio of all-grade treatment-related adverse events calculated by the random effect (RE) model in Group C (PD-1/PD-L1 *vs* Placebo). Subgroup analysis was performed based on PD-1/PD-L1 and tumor types. **(D)** The odds ratio of all-grade treatment-related adverse events calculated by the random effect (RE) model in Group D (PD-1 *vs* PD-1 + CTLA-4). Subgroup analysis was performed based on tumor types. **(E)** The odds ratio of all-grade treatment-related adverse events calculated by the random effect (RE) model in Group E (PD-1 + CTLA-4 *vs* CTLA-4). **(F)** The odds ratio of all-grade treatment-related adverse events calculated by the random effect (RE) model in Group F (PD-1 *vs* CTLA-4).

A lower incidence of any treatment-related adverse events was observed in the PD-1/PD-L1 group as compared to the control group, Group A (OR = 0.29, 95%CI:[0.24, 0.36], I^2^ = 81%, Z = 11.14 (P <0.00001), **Figure 5A**) (18–40). High heterogeneity, through subgroup analyses, was associated with the OSCC and PD-L1 related UC groups (I^2^ = 81%; **Figure 5A**) (18–20, 27). Differences between PD-1 and PD-L1 groups were statistically insignificant (*P* = 0.19; **Supplementary Figure S1C**). Converging trends emerged when the use of PD-1 only was compared with the regimen comprising PD-1 in combination with CTLA-4, in Group D (OR = 0.36, 95%CI:[0.23, 0.56], I^2^ = 54%, Z = 4.56 (P <0.00001); **Figure 5D**) (21, 50, 57, 61). High heterogeneity (I^2^ = 54%), attributed to the lung cancer subgroup, was observed (I^2^ = 59%; **Figure 5D**) (21, 61), but there were no obvious publication bias (**Supplementary Figure S5D**).

Compared to the placebo in Group C (50, 52, 53, 56), PD-1/PD-L1 increased the incidence risk of any treatment-related adverse events with low heterogeneity being observed mainly due to the melanoma subgroup (OR = 1.94, 95%CI:[1.58, 2.38], I^2^ = 13%, Z = 6.41 (*P <*0.00001); **Figure 5C**) (50, 52). There was neither obvious publication bias (**Supplementary Figures S5C, B, E, F** **)** nor statistically significant differences in the subgroup analyses (**Figures 5B, E, F** **)**.

### Incidence of Treatment-Related Grades 3–5 Adverse Events

**Figure 6:**
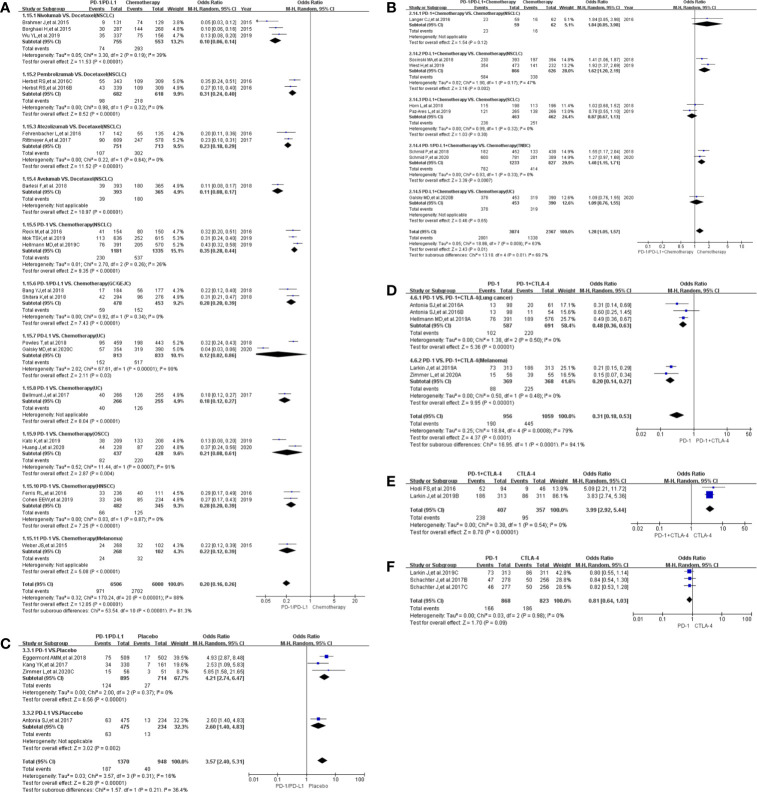
Forest plots of the risk of grades 3–5 treatment-related adverse events **(A)** The odds ratio of grades 3–5 treatment-related adverse events calculated by the random effect (RE) model in Group A (PD-1/PD-L1 *vs* Chemotherapy). Subgroup analysis was performed based on tumor types, PD-1/PD-L1 and treatment regimens. **(B)** The odds ratio of grades 3–5 treatment-related adverse events calculated by the random effect (RE) model in Group B (PD-1/PD-L1 + Chemotherapy *vs* Chemotherapy). Subgroup analysis was performed based on PD-1/PD-L1 and tumor types. **(C)** The odds ratio of grades 3–5 treatment-related adverse events calculated by the random effect (RE) model in Group C (PD-1/PD-L1 *vs* Placebo). Subgroup analysis was performed based on PD-1/PD-L1. **(D)** The odds ratio of grades 3–5 treatment-related adverse events calculated by the random effect (RE) model in Group D (PD-1 *vs* PD-1 + CTLA-4). Subgroup analysis was performed based on PD-1/PD-L1 and tumor types. **(E)** The odds ratio for grades 3–5 of treatment-related adverse events calculated by the random effect (RE) model in Group E (PD-1 + CTLA-4 *vs* CTLA-4). **(F)** The odds ratio for grades 3–5 of treatment-related adverse events calculated by the random effect (RE) model in Group F (PD-1 *vs* CTLA-4).

As observed for any treatment-related adverse events in Group A, the incidence of grades 3–5 adverse events among recipients of PD-1/PD-L1 was significantly lower than for those in the control group [OR = 0.20, 95%CI:(0.16, 0.26), I^2^ = 88%, Z = 12.05 (*P <*0.00001); **Figure 6A**] (18–21, 23–25, 27–30, 32–40). Both OSCC and PD-L1 related UC were determined, through subgroup analysis, to lead to the observed high heterogeneity (I^2^ = 88%) (**Figure 6A**) (18–20, 27). No statistically significant differences were apparent in the risk of grades 3–5 adverse events between either the PD-1 and PD-L1 groups (*P* = 0.19; **Supplementary Figure S1D**) (18–21, 23–25, 27–30, 32–40) or the use of PD-1 alone or in combination with CTLA-4, in Group D [OR = 0.31, 95%CI:(0.18, 0.53), I^2^ = 79%, Z = 4.37 (P <0.00001); **Figure 6D**] (21, 50, 57, 61). The high heterogeneity seen with these groups was inherent to the data and no publication bias was found (**Figure 6D**; **Supplementary Figure S6D**) (21, 50, 57, 61). No statistical analysis results was also found in Group F (**Figure 6F** and **Supplementary Figure S6F**) (57, 63).

When combined with chemotherapy, PD-1/PD-L1 increased the risk of treatment-related grades 3–5 adverse events as compared with the use of chemotherapy alone [OR = 1.28, 95%CI:(1.05, 1.57), I^2^ = 63%, Z = 2.43(P = 0.01); **Figure 6B**] (19, 41, 43–45, 47–49). The overall high heterogeneity (I^2^ = 63%) was traced to the NSCLC subgroup (I^2^ = 47%) (**Figure 6B**) (43, 44). Similar findings were evident in Group E (OR = 3.99, 95%CI: [2.92, 5.44], I^2^ = 0%, Z = 8.70 (P <0.00001), (**Figure 6E**) (57, 58), when PD-1/PD-L1 in combination with CTLA-4 is compared with the sole use of CTLA-4. The corresponding funnel plot are depicted in **Supplementary Figure S6E**.

Finally, compared to the placebo in Group C (50, 52, 53, 56), PD-1/PD-L1 increased the incidence (37, 50, 61, 62, 65, 67) of treatment-related grades 3–5 adverse events with low heterogeneity which was considered to be mainly caused by the PD-L1 related subgroup (OR = 3.57, 95%CI:[2.40, 5.31], I^2^ = 16%, Z = 6.28 (*P <*0.00001); **Figure 6C**) (56). As with other groups, there was no apparent publication bias (**Supplementary Figure S6C**) (50, 52, 53, 56) as also witnessed for Group F featuring the comparison between PD-1 and CTLA-4 (**Figure 5F** and **Supplementary Figure S5F**) (57, 63).

## Discussion

PD-1/PD-L1 inhibitors have been playing an increasingly important role in anti-tumor therapy (1, 5, 6, 8). While these agents have been reported to achieve gratifying clinical anti-tumor efficacy, they are beset by a growing list of diverse treatment-related side effects (18–70). As more clinical trials have been completed in recent years, it is critical that information about the safety and efficacy of PD-1/PD-L1 inhibitors are updated to provide the latest guidance in the administration and use of these therapeutic agents (1, 5, 6, 8, 18–70). The need to provide the most recent information on the safety and adverse effect profiles of PD-1/PD-L1 inhibitors motivated the current meta-analysis.

Following the selection criteria, 44 clinical trials reported by 53 articles were included in the meta-analysis (18–70). High risk of attrition bias was noticeable due to articles with incomplete data (**Figure 2**) (22, 31, 43, 51, 54, 55, 58–60).

Our meta-analysis found that PD-1/PD-L1 inhibitors were generally distinguished in having a more favorable safety profile as compared to chemotherapy, across the four safety indicators applied to the analysis. Similarly, stratified investigation also revealed that between them, PD-L1 inhibitors were associated with fewer cases of adverse events as compare to PD-1 inhibitors, especially when considering the incidences of treatment-related adverse events leading to discontinuation of therapy or death. This observation is contrary to the conclusion reached in the mirror principle based meta-analysis (71). As there lacked randomized controlled trials between PD-1 and PD-L1 (18–70), the differences in the adverse event profiles between these two groups of agents were controversial as well as inconclusive (71). High heterogeneity was found across three evaluation indicators (**Figures 4A**; **5A** and **6A**) and the subgroup analyses suggested the role of the tumor types and the inherent quality of the data in this observation (18–21, 27, 33, 61). Notably, however, there was no obvious publication bias in the articles (**Supplementary Figures S3A**; **S4A**; **S5A** and **S6A**). In addition, the trend in adverse events was repeated when PD-1/PD-L1 inhibitors were compared with combinational use with CTLA-4 (**Figures 3D**; **4D**; **5D** and **6D**) (21, 22, 50, 57–61). The combined results from the above analyses led us to the conclusion that PD-1/PD-L1 inhibitors display better safety characteristics than chemotherapy or the combined use of PD-1/PD-L1 with CTLA-4.

Although PD-1/PD-L1 inhibitors, when prescribed in combination with chemotherapy, increased the occurrence of the four classes of adverse events (**Figures 3B**; **4B**; **5B** and **Figure 6B**) (19, 41–49), the increase was only statistically significant regarding grades 3–5 treatment-related adverse events [OR = 1.28, 95%CI:(1.05, 1.57), I^2^ = 63%, Z = 2.43 (P = 0.01); **6B**] (19, 41, 43–45, 47–49). The high heterogeneity (I^2^ = 63%) was tied to the NSCLC group (I^2^ = 47%; **Figure 6B**) (43, 44). The failure to note any meaningful differences with the other groups (**Figures 3B**; **4B** and **5B**) might be due to the limitation of data. In order to draw more conclusive statistically significant analysis, more clinical trial results need to be considered.

Similar trends in the profile of adverse events were seen when the use of PD-1/PD-L1 inhibitors is compared to placebo (**Figures 3C**; **4C**; **5C** and **6C**) (50–56). We, however, had too few clinical trials to enable us to evaluate the comparisons in the differences in the incidence of treatment-related death [OR = 1.47, 95%CI: (0.34, 6.39), I^2^ = 0%, Z = 0.52 (P = 0.61); **Figure 3C**] (52, 53).

We experienced similar challenges and limitations in the attempt to carry out subgroup analysis based on the treatment regimen and safety indicators, due to insufficient volumes of data. The observed trends and potential differences within the various subgroups need to be verified by using more clinical trials data.

At the time of conducting this study, results from some randomized controlled clinical trials involving PD-1/PD-L1 combined with targeted therapy had also been reported. However, due to the differences among articles and the results obtained, they could not be included in the current meta-analysis. These references were, nonetheless, listed in **Table 1** (21, 22, 26, 36, 42, 50, 61, 63–70).

In summary, our meta-analysis indicates that there is a better safety profile in the use of PD-1/PD-L1 inhibitors as compared to either chemotherapy or the use of combined regimens incorporating PD-1/PD-L1 inhibitors. The PD-1/PD-L1 inhibitors, however, had a worse adverse event profile over placebo. The present study, therefore, suggests caution and awareness of the occurrence of treatment-related adverse events when PD-1/PD-L1 inhibitors are used solely or in combination with other interventions. Clinicians should be aware that should adverse events occur in combinational treatment, withdrawing PD-1/PD-L1 inhibitor may not be the first approach to alleviate severe drug-related toxicities. This meta-analysis provides insights into important considerations to bear in mind when using PD-1/PD-L1 inhibitors and what adverse events to anticipate.

## Conclusion

PD-1/PD-L1 inhibitors display better safety profiles than either chemotherapy or combinational treatment regimens involving PD-1/PD-L1 inhibitors.

## Data Availability Statement

The original contributions presented in the study are included in the article/**Supplementary Material**. Further inquiries can be directed to the corresponding authors.

## Author Contributions

YT, AH, YY, QD, and QW collected the data. YT, AH, YY, and QD performed data cleaning and analysis. YT drafted the manuscript. YS and LW reviewed the manuscript for scientific soundness. All authors contributed to the article and approved the submitted version.

## Funding

This study was funded by the Academic Promotion Program of Shandong First Medical University (2019QL025; YS), Natural Science Foundation of Shandong Province (ZR2019MH042; YS), Jinan Science and Technology Program (201805064; YS), and Postdoctoral Innovation Project of Jinan (YT).

## Conflict of Interest

The authors declare that the research was conducted in the absence of any commercial or financial relationships that could be construed as a potential conflict of interest.

## Supplementary Material

Supplementary Figure 1 Forest plots for group A (PD-1/PD-L1 vs Chemotherapy) **(A)** The odds ratio of treatment-related adverse events leading to death calculated by the random effect (RE) model (PD-1/PD-L1 vs Chemotherapy). Subgroup analysis was conducted based on PD-1/PD-L1. **(B)** The odds ratio of treatment-related adverse events leading to discontinuation calculated by the random effect (RE) model (PD-1/PD-L1 vs Chemotherapy). Subgroup analysis was performed based on PD-1/PD-L1. **(C)** The odds ratio of all-grade treatment-related adverse events calculated by the random effect (RE) model (PD-1/PD-L1 vs Chemotherapy). Subgroup analysis was carried out based on PD-1/PD-L1. **(D)** The odds ratio for grade 3-5 of treatment-related any adverse events calculated by the random effect (RE) model (PD-1/PD-L1 vs Chemotherapy). Subgroup analysis was conducted based on PD-1/PD-L1.

Supplementary Figure 2 Funnel plots for group A (PD-1/PD-L1 vs Chemotherapy) **(A)** The odds ratio of treatment-related adverse events leading to death calculated by the fixed effect (FE) model (PD-1/PD-L1 vs Chemotherapy). Subgroup analysis was performed based on PD-1/PD-L1. **(B)** The odds ratio of treatment-related adverse events leading to discontinuation calculated by the fixed effect (FE) model (PD-1/PD-L1 vs Chemotherapy). Subgroup analysis was carried out based on PD-1/PD-L1. **(C)** The odds ratio of all-grade treatment-related adverse events calculated by the fixed effect (FE) model (PD-1/PD-L1 vs Chemotherapy). Subgroup analysis was conducted based on PD-1/PD-L1. **(D)** The odds ratio of grade 3-5 treatment-related adverse events calculated by fixed effect (FE) model (PD-1/PD-L1 vs Chemotherapy). Subgroup analysis was performed based on PD-1/PD-L1.

Supplementary Figure 3 Funnel plots of treatment-related adverse events leading to death **(A)** The funnel plot of treatment-related adverse events leading to death calculated by the fixed effect (FE) model in Group A (PD-1/PD-L1 vs Chemotherapy). Subgroup analysis was performed based on tumor types. **(B)** The funnel plot of treatment-related adverse events leading to death calculated by the fixed effect (FE) model in Group B (PD-1/PD-L1+Chemotherapy vs Chemotherapy). Subgroup analysis was performed based on tumor types. **(C)** The funnel plot of treatment-related adverse events leading to death calculated by the fixed effect (FE) model in Group C (PD-1/PD-L1 vs Placebo). **(D)** The funnel plot of treatment-related adverse events leading to death calculated by the fixed effect (FE) model in Group D (PD-1 vs PD-1+CTLA-4). Subgroup analysis was performed based on tumor types. **(E)** The funnel plot of treatment-related adverse events leading to death calculated by the fixed effect (FE) model in Group E (PD-1+CTLA-4 vs CTLA-4). **(F)** The funnel plot of treatment-related adverse events leading to death calculated by the fixed effect (FE) model in Group F (PD-1 vs CTLA-4). **(G)** The funnel plot of treatment-related adverse events leading to death calculated by the fixed effect (FE) model in Group G (PD-1/PD-L1 vs PD-1/PD-L1+Chemotherapy).

Supplementary Figure 4 Funnel plots of the risk of treatment-related adverse events leading to discontinuation **(A)** The funnel plot of treatment-related adverse events leading to discontinuation calculated by the fixed effect (FE) model in Group A (PD-1/PD-L1 vs Chemotherapy). Subgroup analysis was performed based on tumor types, PD-1/PD-L1 and treatment regimens. **(B)** The funnel plot of treatment-related adverse events leading to death calculated by the fixed effect (FE) model in Group B (PD-1/PD-L1+Chemotherapy vs Chemotherapy). Subgroup analysis was performed based on PD-1/PD-L1. **(C)** The funnel plot of treatment-related adverse events leading to discontinuation calculated by the fixed effect (FE) model in Group C (PD-1/PD-L1 vs Placebo). Subgroup analysis was performed based on tumor types. **(D)** The funnel plot of treatment-related adverse events leading to discontinuation calculated by the fixed effect (FE) model in Group D (PD-1 vs PD-1+CTLA-4). Subgroup analysis was performed based on tumor types. **(E)** The funnel plot of treatment-related adverse events leading to discontinuation calculated by the fixed effect (FE) model in Group E (PD-1+CTLA-4 vs CTLA-4). **(F)** The funnel plot of treatment-related adverse events leading to discontinuation calculated by the fixed effect (FE) model in Group F (PD-1 vs CTLA-4).

Supplementary Figure 5 Funnel plots of all-grade treatment-related adverse events **(A)** The funnel plot of all-grade treatment-related adverse events calculated by the fixed effect (FE) model in Group A (PD-1/PD-L1 vs Chemotherapy) Subgroup analysis was performed based on tumor types, PD-1/PD-L1 and treatment regimens. **(B)** The funnel plot of all-grade treatment-related adverse events calculated by the fixed effect (FE) model in Group B (PD-1/PD-L1+Chemotherapy vs Chemotherapy). Subgroup analysis was performed based on PD-1/PD-L1 and tumor types. **(C)** The funnel plot for all grade treatment-related adverse events calculated by the fixed effect (FE) model in Group C (PD-1/PD-L1 vs Placebo). Subgroup analysis was performed based on PD-1/PD-L1 and tumor types. **(D)** The funnel plot of all-grade treatment-related adverse events calculated by the fixed effect (FE) model in Group D (PD-1 vs PD-1+CTLA-4). Subgroup analysis was performed based on tumor types. **(E)** The funnel plot of all-grade treatment-related adverse events calculated by the fixed effect (FE) model in Group E (PD-1+CTLA-4 vs CTLA-4). **(F)** The funnel plot of all-grade treatment-related adverse events calculated by the fixed effect (FE) model in Group F (PD-1 vs CTLA-4).

Supplementary Figure 6 Funnel plots of grade 3-5 treatment-related adverse events. **(A)** The funnel plot of grade 3-5 treatment-related adverse events calculated by the fixed effect (FE) model in Group A (PD-1/PD-L1 vs Chemotherapy). Subgroup analysis was performed based on tumor types, PD-1/PD-L1 and treatment regimens. **(B)** The funnel plot of grade 3-5 treatment-related adverse events calculated by the fixed effect (FE) model in Group B (PD-1/PD-L1+Chemotherapy vs Chemotherapy).Subgroup analysis was performed based on PD-1/PD-L1 and tumor types. **(C)** The funnel plot of grade 3-5 treatment-related adverse events calculated by the fixed effect (FE) model in Group C (PD-1/PD-L1 vs Placebo). Subgroup analysis was performed based on PD-1/PD-L1. **(D)** The funnel plot of grade 3-5 treatment-related adverse events calculated by the fixed effect (FE) model in Group D (PD-1 vs PD-1+CTLA-4). Subgroup analysis was performed based on PD-1/PD-L1 and tumor types. **(E)** The funnel plot of grade 3-5 treatment-related adverse events calculated by the fixed effect (FE) model in Group E (PD-1+CTLA-4 vs CTLA-4). **(F)** The funnel plot of grade 3-5 treatment-related adverse events calculated by the fixed effect (FE) model in Group F (PD-1 vs CTLA-4).

## Abbreviations

PRISMA, Preferred Reporting Items for Systematic Reviews and Meta-Analyses; PICOS, Participants, Interventions, Comparisons, Outcomes, and Study design; PD-1, Programmed Cell Death-1; PD-L1, Programmed Cell Death Ligand 1; CTLA-4, Cytotoxic T lymphocyte associate protein-4; OR, Odds Ratio; CI, Confidence Interval; RE, Random Effect; NSCLC, Non-Small Cell Lung Cancer; SCLC, Small Cell Lung Cancer; OSCC, Oesophageal Squamous Cell Carcinoma; HNSCC, Head and Neck Squamous Cell Carcinoma; UC, Urothelial Cancer; TNBC, Triple-negative Breast Cancer; RCC, Renal Cell Carcinoma; GC/GEJC, Gastric or Gastro-oesophageal Junction Cancer.
