# Supplementary figures and images for: Assessment of the Clinical Trials Safety Profile of PD-1/PD-L1 Inhibitors Among Patients With Cancer: An Updated Systematic Review and Meta-Analysis
Source: Front Oncol. 2021 May 24;11:662392. doi: 10.3389/fonc.2021.662392 (PMC8184020; doi:10.3389/fonc.2021.662392)

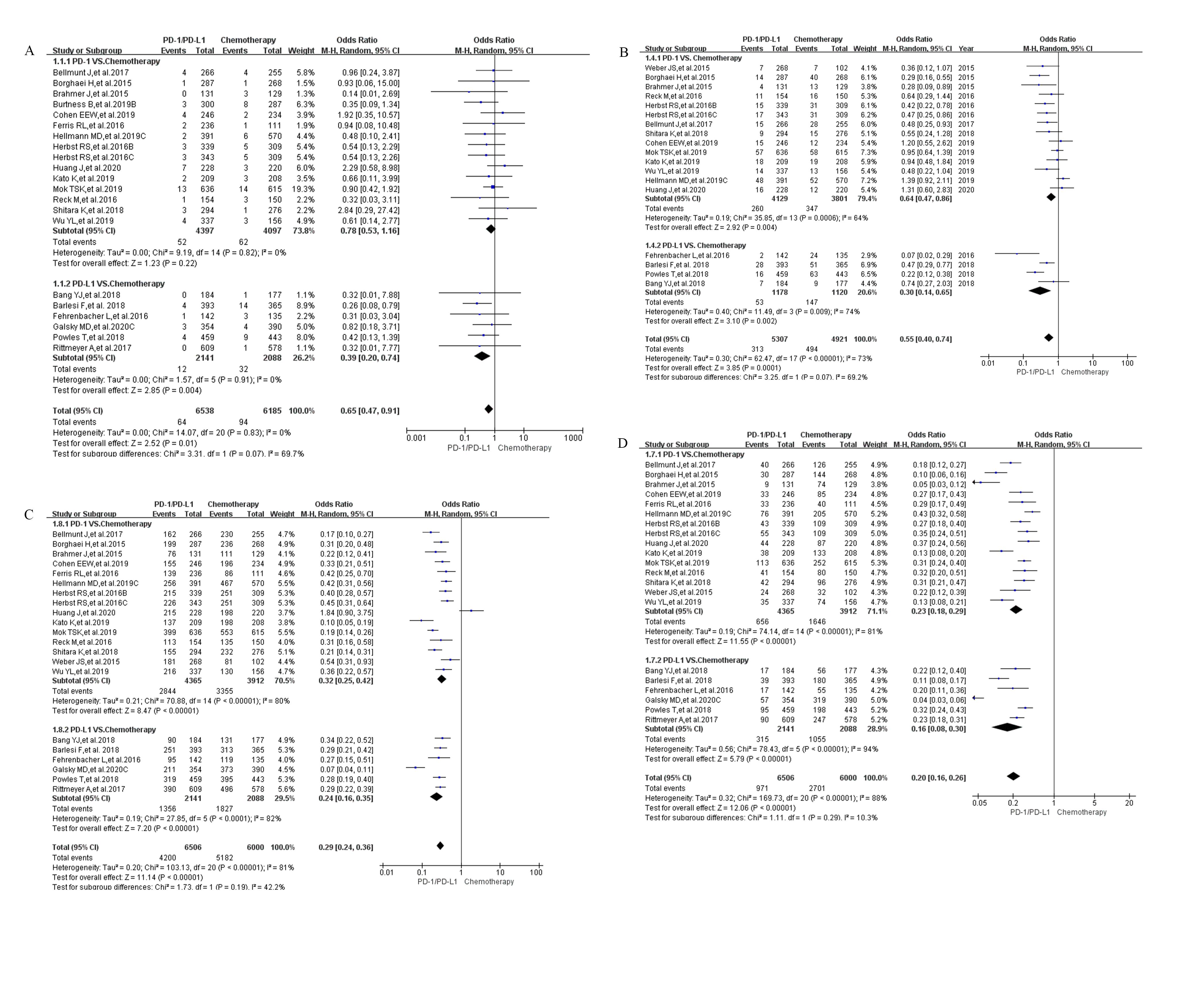

Supplement: Supplementary Figure 1 — Forest plots for group A (PD-1/PD-L1 vs Chemotherapy) (A) The odds ratio of treatment-related adverse events leading to death calculated by the random effect (RE) model (PD-1/PD-L1 vs Chemotherapy). Subgroup analysis was conducted based on PD-1/PD-L1. (B) The odds ratio of treatment-related adverse events leading to discontinuation calculated by the random effect (RE) model (PD-1/PD-L1 vs Chemotherapy). Subgroup analysis was performed based on PD-1/PD-L1. (C) The odds ratio of all-grade treatment-related adverse events calculated by the random effect (RE) model (PD-1/PD-L1 vs Chemotherapy). Subgroup analysis was carried out based on PD-1/PD-L1. (D) The odds ratio for grade 3-5 of treatment-related any adverse events calculated by the random effect (RE) model (PD-1/PD-L1 vs Chemotherapy). Subgroup analysis was conducted based on PD-1/PD-L1. [file Image_1.tif]

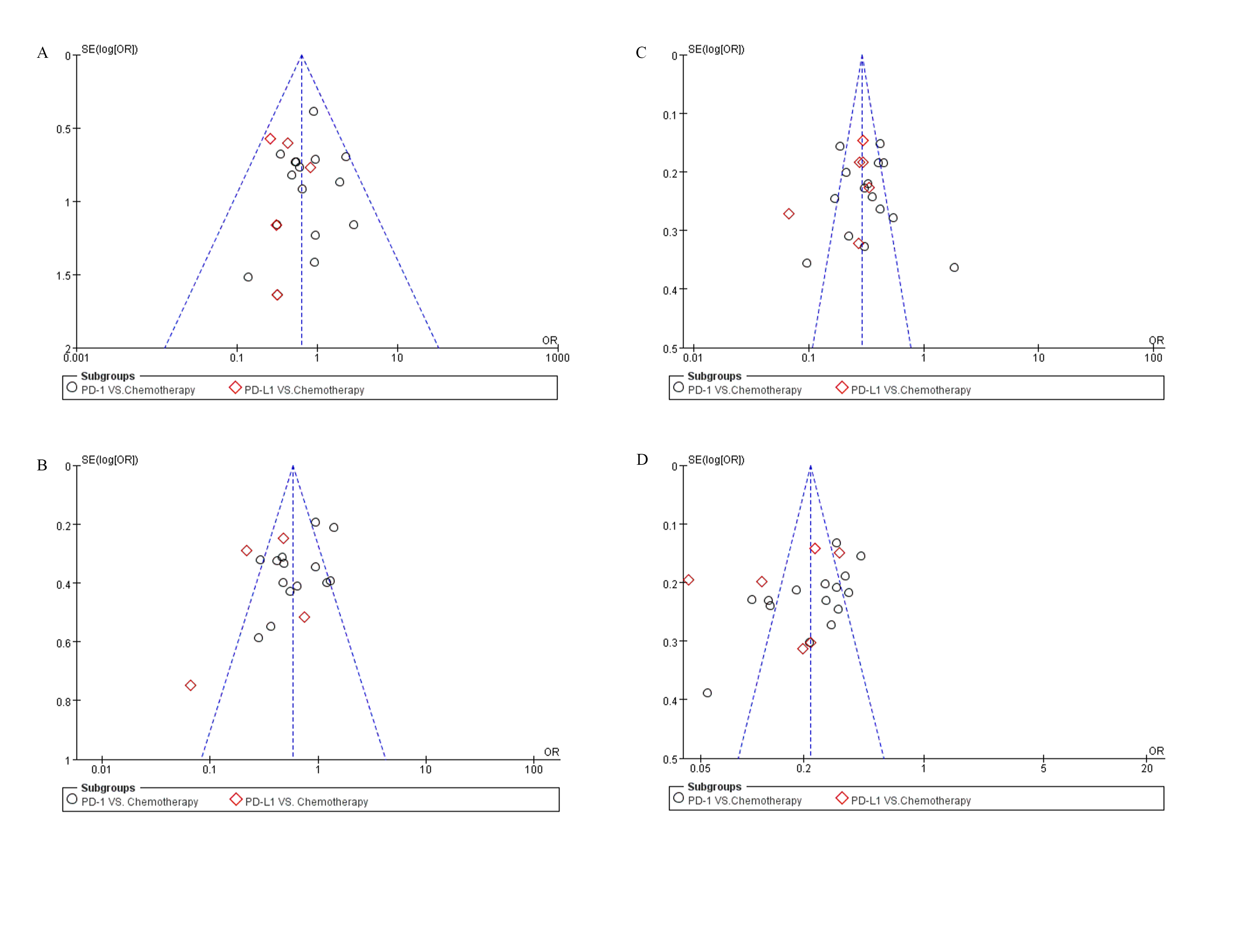

Supplement: Supplementary Figure 2 — Funnel plots for group A (PD-1/PD-L1 vs Chemotherapy) (A) The odds ratio of treatment-related adverse events leading to death calculated by the fixed effect (FE) model (PD-1/PD-L1 vs Chemotherapy). Subgroup analysis was performed based on PD-1/PD-L1. (B) The odds ratio of treatment-related adverse events leading to discontinuation calculated by the fixed effect (FE) model (PD-1/PD-L1 vs Chemotherapy). Subgroup analysis was carried out based on PD-1/PD-L1. (C) The odds ratio of all-grade treatment-related adverse events calculated by the fixed effect (FE) model (PD-1/PD-L1 vs Chemotherapy). Subgroup analysis was conducted based on PD-1/PD-L1. (D) The odds ratio of grade 3-5 treatment-related adverse events calculated by fixed effect (FE) model (PD-1/PD-L1 vs Chemotherapy). Subgroup analysis was performed based on PD-1/PD-L1. [file Image_2.tif]

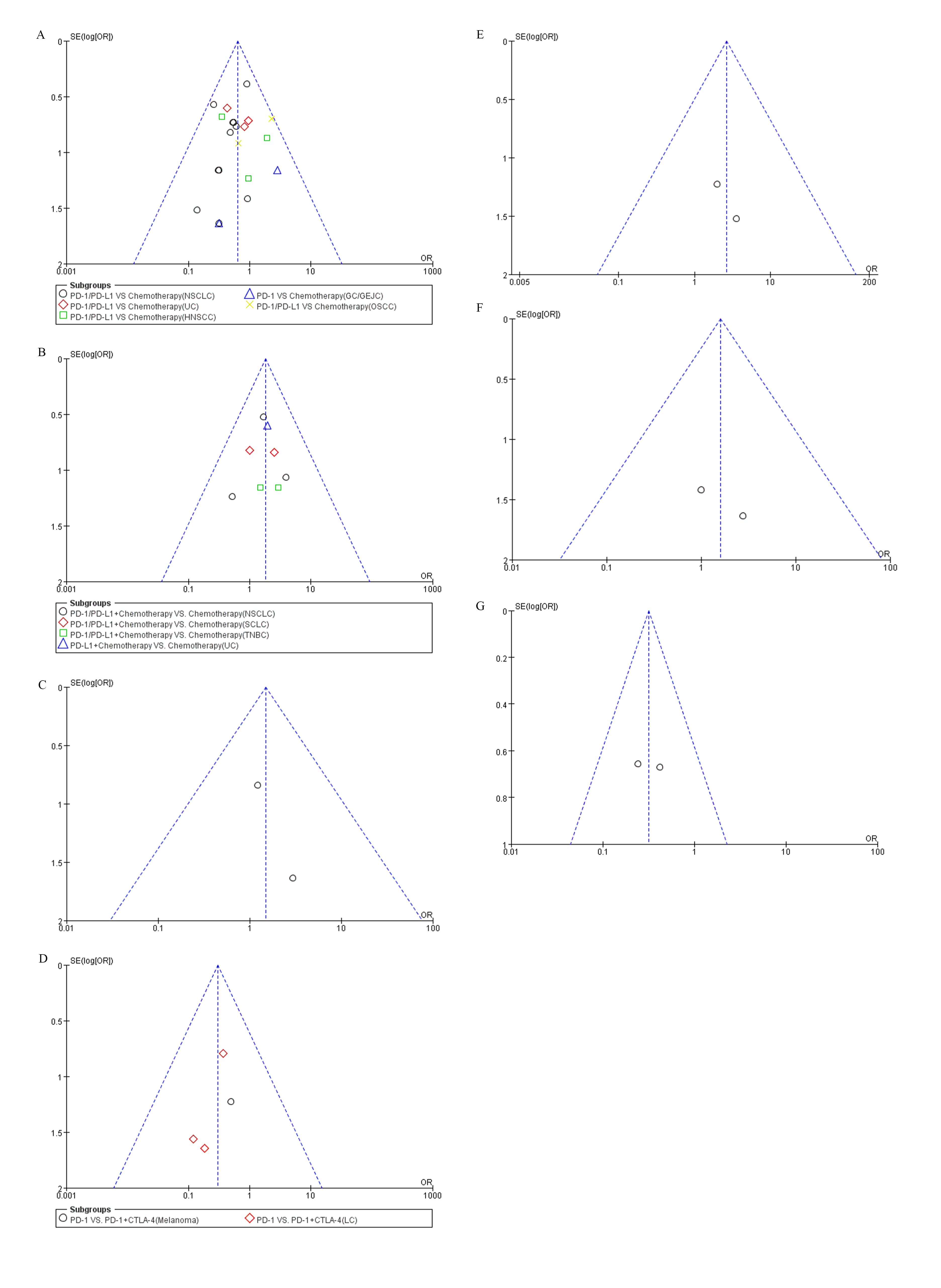

Supplement: Supplementary Figure 3 — Funnel plots of treatment-related adverse events leading to death (A) The funnel plot of treatment-related adverse events leading to death calculated by the fixed effect (FE) model in Group A (PD-1/PD-L1 vs Chemotherapy). Subgroup analysis was performed based on tumor types. (B) The funnel plot of treatment-related adverse events leading to death calculated by the fixed effect (FE) model in Group B (PD-1/PD-L1+Chemotherapy vs Chemotherapy). Subgroup analysis was performed based on tumor types. (C) The funnel plot of treatment-related adverse events leading to death calculated by the fixed effect (FE) model in Group C (PD-1/PD-L1 vs Placebo). (D) The funnel plot of treatment-related adverse events leading to death calculated by the fixed effect (FE) model in Group D (PD-1 vs PD-1+CTLA-4). Subgroup analysis was performed based on tumor types. (E) The funnel plot of treatment-related adverse events leading to death calculated by the fixed effect (FE) model in Group E (PD-1+CTLA-4 vs CTLA-4). (F) The funnel plot of treatment-related adverse events leading to death calculated by the fixed effect (FE) model in Group F (PD-1 vs CTLA-4). (G) The funnel plot of treatment-related adverse events leading to death calculated by the fixed effect (FE) model in Group G (PD-1/PD-L1 vs PD-1/PD-L1+Chemotherapy). [file Image_3.tif]

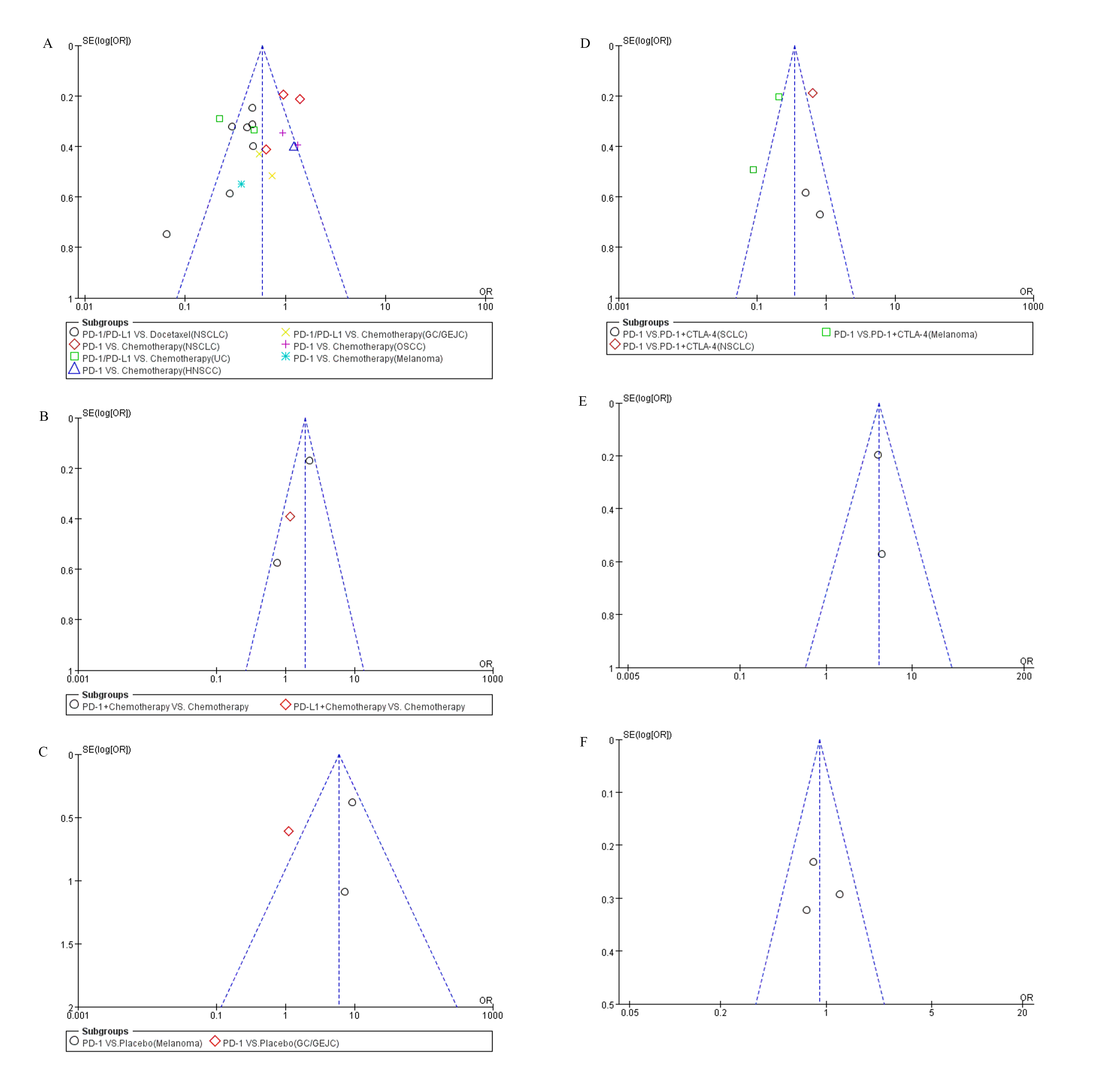

Supplement: Supplementary Figure 4 — Funnel plots of the risk of treatment-related adverse events leading to discontinuation (A) The funnel plot of treatment-related adverse events leading to discontinuation calculated by the fixed effect (FE) model in Group A (PD-1/PD-L1 vs Chemotherapy). Subgroup analysis was performed based on tumor types, PD-1/PD-L1 and treatment regimens. (B) The funnel plot of treatment-related adverse events leading to death calculated by the fixed effect (FE) model in Group B (PD-1/PD-L1+Chemotherapy vs Chemotherapy). Subgroup analysis was performed based on PD-1/PD-L1. (C) The funnel plot of treatment-related adverse events leading to discontinuation calculated by the fixed effect (FE) model in Group C (PD-1/PD-L1 vs Placebo). Subgroup analysis was performed based on tumor types. (D) The funnel plot of treatment-related adverse events leading to discontinuation calculated by the fixed effect (FE) model in Group D (PD-1 vs PD-1+CTLA-4). Subgroup analysis was performed based on tumor types. (E) The funnel plot of treatment-related adverse events leading to discontinuation calculated by the fixed effect (FE) model in Group E (PD-1+CTLA-4 vs CTLA-4). (F) The funnel plot of treatment-related adverse events leading to discontinuation calculated by the fixed effect (FE) model in Group F (PD-1 vs CTLA-4). [file Image_4.tif]

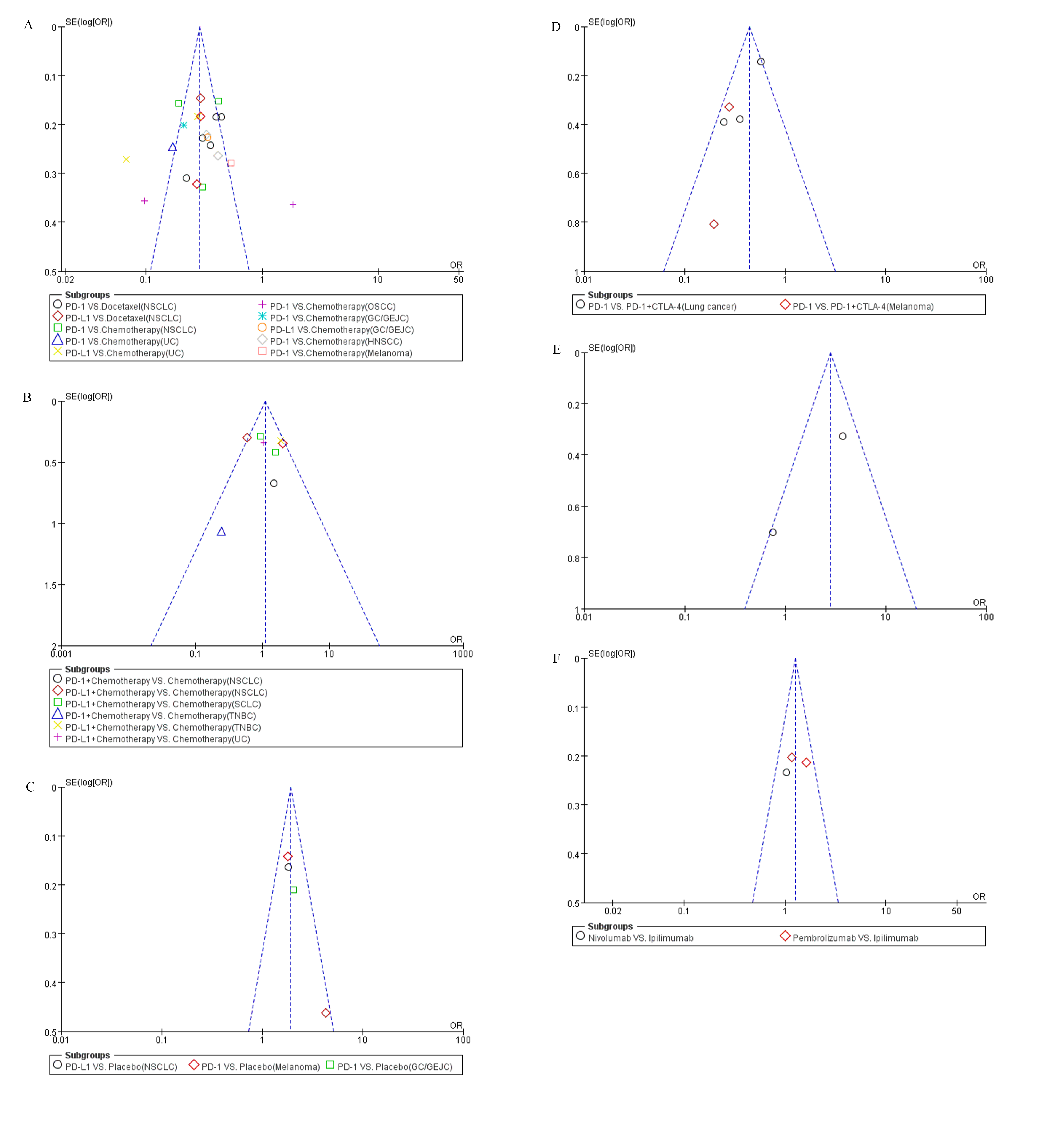

Supplement: Supplementary Figure 5 — Funnel plots of all-grade treatment-related adverse events (A) The funnel plot of all-grade treatment-related adverse events calculated by the fixed effect (FE) model in Group A (PD-1/PD-L1 vs Chemotherapy) Subgroup analysis was performed based on tumor types, PD-1/PD-L1 and treatment regimens. (B) The funnel plot of all-grade treatment-related adverse events calculated by the fixed effect (FE) model in Group B (PD-1/PD-L1+Chemotherapy vs Chemotherapy). Subgroup analysis was performed based on PD-1/PD-L1 and tumor types. (C) The funnel plot for all grade treatment-related adverse events calculated by the fixed effect (FE) model in Group C (PD-1/PD-L1 vs Placebo). Subgroup analysis was performed based on PD-1/PD-L1 and tumor types. (D) The funnel plot of all-grade treatment-related adverse events calculated by the fixed effect (FE) model in Group D (PD-1 vs PD-1+CTLA-4). Subgroup analysis was performed based on tumor types. (E) The funnel plot of all-grade treatment-related adverse events calculated by the fixed effect (FE) model in Group E (PD-1+CTLA-4 vs CTLA-4). (F) The funnel plot of all-grade treatment-related adverse events calculated by the fixed effect (FE) model in Group F (PD-1 vs CTLA-4). [file Image_5.tif]

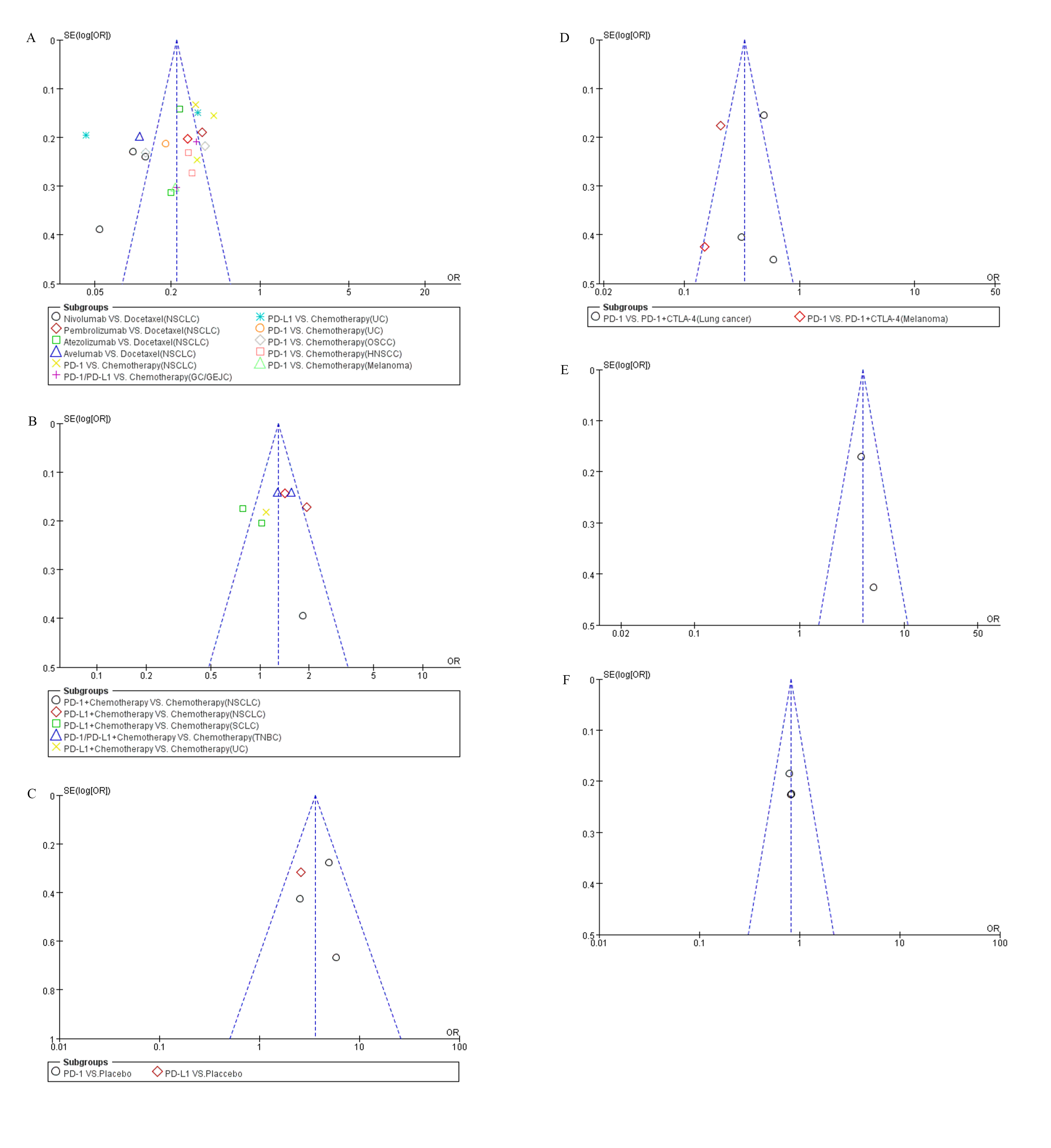

Supplement: Supplementary Figure 6 — Funnel plots of grade 3-5 treatment-related adverse events. (A) The funnel plot of grade 3-5 treatment-related adverse events calculated by the fixed effect (FE) model in Group A (PD-1/PD-L1 vs Chemotherapy). Subgroup analysis was performed based on tumor types, PD-1/PD-L1 and treatment regimens. (B) The funnel plot of grade 3-5 treatment-related adverse events calculated by the fixed effect (FE) model in Group B (PD-1/PD-L1+Chemotherapy vs Chemotherapy).Subgroup analysis was performed based on PD-1/PD-L1 and tumor types. (C) The funnel plot of grade 3-5 treatment-related adverse events calculated by the fixed effect (FE) model in Group C (PD-1/PD-L1 vs Placebo). Subgroup analysis was performed based on PD-1/PD-L1. (D) The funnel plot of grade 3-5 treatment-related adverse events calculated by the fixed effect (FE) model in Group D (PD-1 vs PD-1+CTLA-4). Subgroup analysis was performed based on PD-1/PD-L1 and tumor types. (E) The funnel plot of grade 3-5 treatment-related adverse events calculated by the fixed effect (FE) model in Group E (PD-1+CTLA-4 vs CTLA-4). (F) The funnel plot of grade 3-5 treatment-related adverse events calculated by the fixed effect (FE) model in Group F (PD-1 vs CTLA-4). [file Image_6.tif]
